# Novel Gene Rearrangement Pattern in *Pachycrepoideus vindemmiae* Mitochondrial Genome: New Gene Order in Pteromalidae (Hymenoptera: Chalcidoidea)

**DOI:** 10.3390/ani13121985

**Published:** 2023-06-14

**Authors:** Yixin Huang, Yuanhan Yang, Liqing Qi, Haoyuan Hu, Jean-Yves Rasplus, Xu Wang

**Affiliations:** 1 Collaborative Innovation Center of Recovery and Reconstruction of Degraded Ecosystem in Wanjiang Basin Co-founded by Anhui Province and Ministry of Education, School of Ecology and Environment, Anhui Normal University, Wuhu 241000, China; 2 Key Laboratory of Zoological Systematics and Evolution, Institute of Zoology, Chinese Academy of Sciences, Chaoyang District, Beijing 100101, China; 3 Centre de Biologie pour la Gestion des Populations (CBGP), INRAE, CIRAD, IRD, Montpellier SupAgro, Université de Montpellier, 34398 Montpellier, France; 4 Anhui Provincial Key Laboratory of the Conservation and Exploitation of Biological Resources, College of Life Sciences, Anhui Normal University, Wuhu 241000, China

**Keywords:** Pteromalidae, mitogenome, parasitic lifestyles, phylogenetic position, gene rearrangement

## Abstract

**Simple Summary:**

The mitochondrial genome is a reliable genetic marker for reconstructing phylogeny and Pteromalidae is a diverse and complex family of chalcid wasps, but its evolutionary history is still poorly understood. In this study, we sequenced the mitochondrial genomes of four species (Muscidifurax similadanacus, M. sinesensilla, Nasonia vitripennis, and Pachycrepoideus vindemmiae) of Pteromalidae. Additionally, a phylogenetic hypothesis was reconstructed for the subfamilies of Pteromalidae that includes newly acquired mitogenomes and those deposited in NCBI. We used pairwise breakpoint distances to infer this phylogeny. Our study enriches the overall knowledge on gene rearrangement in Pteromalidae, reveals the evolutionary relationships among several major groups of Pteromalidae, accumulates molecular data for a Pteromalidae phylogeny, and provides a genetic background basis for biological control in agriculture and forestry.

**Abstract:**

The mitochondrial genomes of *Muscidifurax similadanacus*, *M. sinesensilla*, *Nasonia vitripennis*, and *Pachycrepoideus vindemmiae* were sequenced to better understand the structural evolution of Pteromalidae mitogenomes. These newly sequenced mitogenomes all contained 37 genes. Nucleotide composition was AT-biased and the majority of the protein-coding genes exhibited a negative AT skew. All 13 protein-coding genes (PCGs) initiated with the standard start codon of ATN, excepted for *nad1* of *N. vitripennis*, which started with TTG, and terminated with a typical stop codon TAA/TAG or an incomplete stop codon T. All transfer RNA (tRNA) genes were predicted to fold into the typical clover-leaf secondary structures, except for *trnS1*, which lacks the DHU arm in all species. In *P. vindemmiae*, *trnR* and *trnQ* lack the DHU arm and TΨC arm, respectively. Although most genes evolved under a strong purifying selection, the Ka/Ks value of the *atp8* gene of *P. vindemmiae* was greater than 1, indicating putative positive selection. A novel transposition of *trnR* in *P. vindemmiae* was revealed, which was the first of this kind to be reported in Pteromalidae. Two kinds of datasets (PCG12 and AA) and two inference methods (maximum likelihood and Bayesian inference) were used to reconstruct a phylogenetic hypothesis for the newly sequenced mitogenomes of Pteromalidae and those deposited in GenBank. The topologies obtained recovered the monophyly of the three subfamilies included. Pachyneurinae and Pteromalinae were recovered as sister families, and both appeared sister to Sycophaginae. The pairwise breakpoint distances of mitogenome rearrangements were estimated to infer phylogeny among pteromalid species. The topology obtained was not totally congruent with those reconstructed using the ML and BI methods.

## 1. Introduction

The animal mitochondrial genome is maternally inherited and fast-evolving [1]. Mitochondrial DNA is usually double-stranded and composed of 37 genes, 13 protein-coding genes, 2 rRNAs, and 22 tRNAs [2,3,4]. Information contained by the gene organization of mitogenomes is a valuable tool for phylogenetic and genetic studies [5,6,7,8,9]. Moreover, mitochondrial genome sequences are widely used in studies of molecular evolution and population genetics [10,11,12,13,14]. In recent years, an increasing number of mitochondrial genomes have been sequenced in Hymenoptera with a phylogenetic purpose.

The phylogeny of Pteromalidae has long been debated and is still controversial. The relationships among Pteromalidae and other chalcid families are still uncertain. Chen et al. considered that Agaonidae was closely related to Pteromalidae [15], whereas Wu et al. proposed that Pteromalidae and Eurytomidae were sister groups [16]. Pteromalidae has been considered as a polyphyletic group for decades [17]. This concept was supported by several studies [18,19,20], even though Pteromalidae was recovered as monophyletic in several other studies [16,21,22]. Rasplus et al. [23] suggested that Sycoecinae, Otitesellinae, and Sycoryctinae (used to be Agaonidae) should be transferred to Pteromalidae based on ribosomal and mitochondrial genes as well as morphology. Dzhanokmen [24] combined morphology and biological characters to reconstruct an unformal phylogeny and suggested Pteromalinae to be sister to all other Pteromalidae. Desjardins et al. [25] used four nuclear coding genes to reconstruct the phylogenetic relationship of Pteromalidae. Their results confirmed the monophyly of nearly all tribes represented by multiple specimens, and two subfamilies. Other subfamilies appeared para- or polyphyletic, and monophyly was significantly rejected for Miscogasterinae, Ormocerinae, and Colotrechninae. Munro et al. [18] conducted a molecular analysis based on 18S and 28S ribosomal genes, which supported the monophyly of seven subfamilies and the paraphyly or polyphyly of nine subfamilies. A similar result was recovered by Heraty et al. [19]. Pteromalidae, as historically understood, is both a highly diverse and a highly disparate group, these properties rendering it difficult to reconstruct a reliable phylogenetic hypothesis based on both morphological and molecular characters. Hereafter, we propose to investigate the relationships among Pteromalidae based on complete mitochondrial genomes.

In most insect groups, gene rearrangements in mitogenomes are relatively rare events and can be reliably used as phylogenetic markers [3]. To the opposite, gene rearrangements appear common in Chalcidoidea mitogenomes and may represent evolutionarily independent events. Gene rearrangements in Chalcidoidea have been hypothesized to correlate with the diversity of their life-history traits and with their phylogenetic positions [14]. In Pteromalidae, twenty nearly complete mitochondrial genomes belonging to four subfamilies have been reported so far, and a total of 14 gene rearrangements have been found in these subfamilies [14,16,26]. Oliveira et al. [26] first sequenced a partial mitochondrial genome of *N. vitripennis*. Compared with a hypothetical ancestral pattern, they identified six inverted protein-coding genes (PCGs). The incomplete mitochondrial genome of *Nasonia vitripennis* Walker, 1836 (Pteromalidae) was the first mitochondrial genome to be reported in Chalcidoidea; it showed novel gene rearrangements compared to other insects and was considered the typical model for Chalcidoidea [26]. However, nine genes were not sequenced and the gene arrangements of *N. vitripennis* were still partly undetermined. Subsequently, Xiao et al. [14] sequenced the mitochondrial genomes of two species of Sycoryctinae and showed that gene rearrangements mostly concerned tRNAs. Finally, Tang et al. and Wu et al. [16,27] performed mitochondrial sequencing of Pteromalidae species and summarized gene rearrangements.

The CREx program online is widely used to heuristically determine pairwise rearrangement events in mitochondrial genomes [28]. It considers transpositions, reverse transpositions, reversals, and tandem-duplication-random-loss (TDRL) events, which are based on common intervals that reflect genes that appear consecutively in several of the input gene orders. Wu et al. [16] proposed that “closely related groups in lower taxonomic levels tend to exhibit more similar gene orders, which indicates that gene rearrangements may still be useful for phylogenetic analysis at higher taxonomic levels. Pairwise breakpoint distances can be used to analyze the rates of mitochondrial gene rearrangement for Chalcidoidea using the CREx web server”.

We measured complete or nearly complete mitochondrial genomes of four Pteromalidae, *Muscidifurax similadanacus* Xiao and Zhou, 2018 [29], *M. sinesensilla* Xiao & Zhou, 2018 [29], and *N. vitripennis* as primary parasitoids and *Pachycrepoideus vindemmiae* Rondani, 1875 which is both a primary parasitoid and hyperparasitoid [29,30,31,32,33,34,35,36]. Using these newly acquired mitogenomes of Pteromalidae as well as those deposited in GenBank, we propose a phylogenetic hypothesis based on mitogenomes for Pteromalidae. A second aim of this study is to analyze the characteristics of the Pteromalidae mitogenomes and to test the ability of pairwise breakpoint distances to infer reliable phylogenetic relationships.

## 2. Materials and Methods

### 2.1. Sample Preparation and DNA Extraction

All specimens were collected from the wild, including the three primary parasitoids and *Pachycrepoideus vindemmiae* as primary parasitoids and hyperparasitoids [37]. *M. similadanacus* and *M. sinesensilla* were collected in Xinjiang province of China, *N. vitripennis* was collected in Shandong province, and *P. vindemmiae* in Anhui province (Table 1), and then they were reared in the laboratory for more than three years. All the specimens were stored at −20 °C in absolute ethanol prior to DNA extraction. Total genomic DNA was extracted using the cetyltrimethyl ammonium bromide (CTAB) method [38].

### 2.2. Sequencing and Assembly

Sequencing was performed using a whole-genome shotgun (WGS) strategy on the Illumina Novaseq platform. The PCR library was constructed with the use of a TruSeqTM DNA Sample Prep Kit for each species using genomic DNA with an insert size of 350 bp and was sequenced on the Illumina platform at Berry Genomics, Beijing; a total of 10 Gb clean data (150 bp pair-end reads) were obtained for each species. The fragments were repaired by the combined action of 3′-5′ exonuclease and polymerase in an End Repair Mix. Thereafter, magnetic beads purified the connection products, which were incubated with the DNA fragment to remove free and self-connecting joint sequences. Finally, the homogenized and mixed libraries were gradually diluted and quantified to 4–5 pM for sequencing. The quality of data was checked with FastQC [6,41]. The original data adapter had been removed by AdapterRemoval version 2 [42]. SOAPec version 2.01 was used for quality correction with K-mer set to 17. Reads with a length of less than 50 bp were excluded. Geneious v 11.0.2 was used to assemble and annotate the mitochondrial (mt) genomes [6,43]. The COX1 gene of *N. vitripennis* (MT635402) was used as a reference to map and to identify the COX1 gene of these four pteromalid species in our mitogenome sequences [44]. Subsequently, the sequence was extended on each side using a de novo method based on the local alignment method.

### 2.3. Mitochondrial Genome Annotation

The tRNA genes and protein-coding genes were identified using the MITOS Webserver. The secondary structure was also predicted using the MITOS WebSever, setting the parameters with the Invertebrate Mito genetic code [6,45]. Protein-coding genes (PCGs) were identified as open reading frames corresponding to the 13 PCGs in the metazoan mitogenomes. Mitogenome maps were produced using Organellar Genome DRAW (OGDRAW) [46].

### 2.4. Comparative Analysis

Base composition and relative synonymous codon usage (RSCU) were analyzed using MEGA X [47]. Geneious v 11.0.2 [43] was used to check all the genes in the mitochondrial genome. Comparative analyses of codon usage for these four mitogenomes were calculated using PhyloSuite [22,45]. The predicted secondary structures of all tRNAs were drawn using Adobe Illustrator CC 2018. Multiple-substitution correction of Non-synonymous (Ka)/synonymous (Ks) mutation rate ratios among the 13 PCGs was calculated with DnaSP v5, using that of *Pachyneuron aphidis* (Pteromalidae: Pachyneurinae) as a reference sequence [48,49]. AT/GC skewness was calculated as AT skew= (A − T)/(A + T) and GC skew = (G − C)/(G + C) [50].

### 2.5. Phylogenetic Analysis

To reconstruct the phylogeny of Pteromalidae, 4 newly generated mitogenomes and 18 others downloaded from GenBank were analyzed. Species of *Eupelmus* (Eupelmidae) and *Torymus* (Torymidae) were used as outgroups (Table 1). All protein-coding genes were aligned individually based on codon optimized multiple alignments using the MAFFT 7.3.1 and G-INS-I algorithm [51]. Aligned sequences were then concatenated into two different datasets (PCG12, including the first and second codon of 13 protein-coding genes, and AA: 13 PCGs translated into amino acids). Maximum-likelihood (ML) analysis was conducted in W-IQtree [52] using the best-fit substitution model. An ultrafast bootstrap (UFB) [53] of 1000 replications and the SH-aLRT test [54] was used to assess branch supports. Bayesian inference was implemented in PhyloBayes MPI 1.5a [55] and we used a site-heterogeneous mixture model (CAT+GTR). The trees were sampled every 1000 generations, and the burning rate was set as 0.25 of the sampled value. FigTree v.1.3.1 [56] was used to view topologies.

### 2.6. Ancestral Character Reconstruction and Gene Rearrangement in Pteromalidae

To explore the effect of parasitic lifestyles (primary parasitoids or/and hyperparasitoids) and of the phylogenetic position in gene rearrangement, we performed an ancestral character reconstruction using Mesquite 3.04 [57]. Because some mitogenome sequences were incomplete and pairwise breakpoint distances could not be calculated, we used mitogenomes with 37 genes (13 PCGs, 2 rRNAs, 22 tRNAs) and the complete/incomplete control region to performed our test.

Four newly generated mitogenomes and three obtained from GenBank were analyzed to reconstruct the ancestral character of Pteromalidae. The parasitic lifestyle of the species included in our dataset was coded as follows: 1: primary parasitoids; 2: both primary parasitoids and hyperparasitoids; and 3: undefined. The results obtained were organized in figures using Adobe Illustrator CC 2018. A map of the mitochondrial gene rearrangement was depicted with the Illustrator of biological sequences (IBS) [58]. Pairwise breakpoint distances (PBD) were used to analyze the rate of mitochondrial gene rearrangement for Pteromalidae (complete mitogenomes only) using the CREx web server [28]. A heatmap of the pairwise breakpoint distance was drawn using TBtools [59].

## 3. Results

### 3.1. Genome Structure

**Figure 1:**
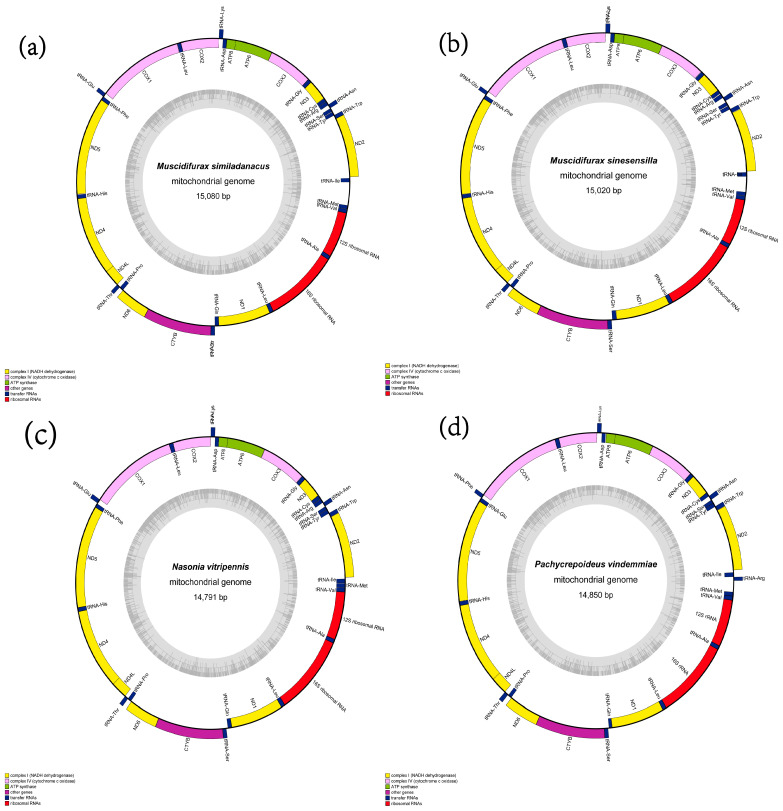
Circular maps of the mitochondrial genomes of *M. similadanacus*, *M. sinesensilla*, *N. vitripennis*, and *P. vindemmiae* of Pteromalidae. Protein-coding and ribosomal genes are shown with standard abbreviations. The N strand is visualized on the outer circle and the J strand on the inner circle. (**a**) Circular map of the mitochondrial genome of *M. similadanacus*. (**b**) Circular map of the mitochondrial genome of *M. sinesensilla*. (**c**) Circular map of the mitochondrial genome of *N. vitripennis*. (**d**) Circular map of the mitochondrial genome of *P. vindemmiae*.

The *M. similadanacus* (GenBank accession number: MT712139), *M. sinesensilla* (GenBank accession number: MT712140), *N. vitripennis* (GenBank accession number: MT712141), and *P. vindemmiae* (GenBank accession number: MT712142) mitochondrial genomes have a total length of 15,080 bp, 15,020 bp, 14,791 bp, and 14,850 bp, respectively, and all were typical double-chain circular molecular structures (Figure 1). They contained 22 tRNA genes, 2 rRNA genes (*rrnL* and *rrnS*), and 13 protein-coding genes (*nad1-6*, *nad4l*, *cox1-3*, *atp8*, *atp6*, *cytb*). The transcription direction of encoding genes observed in the newly sequenced mitogenomes was generally consistent with other insects. The positions of four genes (*trnR*, *trnF*, *trnN*, *trnE*) of *P. vindemmiae* differ from those for the three other newly sequenced species (Table 2 and Table 3). In *P. vindemmiae*, 28 genes (16 tRNAs, 10 PCGs, and 2 rRNAs) were in the J chain, and 9 genes (6 tRNAs and 3 PCGs) and an AT-rich region were located in the N chain.

### 3.2. Characteristics of Base Composition

The nucleotide composition of the mitogenomes exhibits a high A + T content, with an average of 83.98%, which is usual in mtDNA in arthropods and Hymenoptera [60,61,62]. Among the newly generated mitogenomes, *P. vindemmiae* had the highest AT content (85.3%) and *N. vitripennis* had the lowest (83.0%) (Table 1 and Table 2). Based on the analysis of base content, the range of the AT (GC) skew value varies from 0.018 (−0.112) to 0.047 (−0.091); for *P. vindemmiae* it was 0.036 (−0.108), indicating a high A-T bias in Pteromalidae mitogenomes.

Among the 13 PCGs, the highest AT content was 84.2% for *P. vindemmiae*, and the lowest was 81.7% for *N. vitripennis*. Among the newly generated mitogenomes, *P. vindemmiae* had the highest AT contents for the full genome, PCGs, tRNAs, and rRNAs, which were 85.3%, 84.2%, 88.9%, and 88.0%, respectively (Table 4). There are three hydrogen bonds between G and C and two hydrogen bonds between A and T, so GC was more stable than AT. High AT content is likely to be one of the most important factors explaining rearrangement in mitochondrial genomes.

### 3.3. Overlap and Gap

A total length of 370 bp of gaps were found in the newly sequenced mitogenomes, of which *M. similadanacus* had 58 bp of gaps, *M. sinesensilla* 121 bp, *N. vitripennis* 94 bp, and *P. vindemmiae* 97 bp. Among these species, the longest gap was 35 bp and was located between *trnI* and *nad2* of *P. vindemmiae*. The shortest gap was 1 bp and was located between *trnQ* and *nad1* of *M. similadanacus, trnN* and *trnR* and *trnH* and *nad4* of *M. sinesensilla*, *trnD* and *atp8* and *cytb* and *nad6* with *N. vitripennis, trnS1* and *trnY* and *trnC* and *trnN*, *cox2*, and *trnK*, *trnQ*, and *nad1* of *P. vindemmiae*.

Overlapping genes are common in mitochondrial genomes of arthropods. A total of 98 bp of overlaps were observed in our sequenced mitogenomes. Overlapping genes might reflect the selection for a short and economic mitogenome, and they usually involve trn genes, because their sequences are constrained by fewer mutations [63,64]. Most of the discovered overlaps appeared in tRNA. *M. similadanacus* had 31 bp of overlaps, *M. sinesensilla* 23 bp, *N. vitripennis* 21 bp, and *P. vindemmiae* 23 bp. The length of overlap ranged from 1 to 10 bp, and the longest was located between *atp8* and *atp6* of *M. similadanacus* and *M. sinesensilla*. The shortest was 1 bp and were located between *atp6* and *cox3*, *cytb* and *nad6*, and *trnS2* and *cytb* of *M. similadanacus*; *nad3* and *trnC* and *atp6* and *cox3* of *M. sinesensilla*; *nad3* and *trnC* and *nad5* and *trnF* of *N. vitripennis*; and *atp6* and *cox3*, *trnT* and *nad4l*, and *cytb* and *nad6* of *P. vindemmiae*.

### 3.4. Transfer RNA and Ribosomal RNA Genes

**Figure 2:**
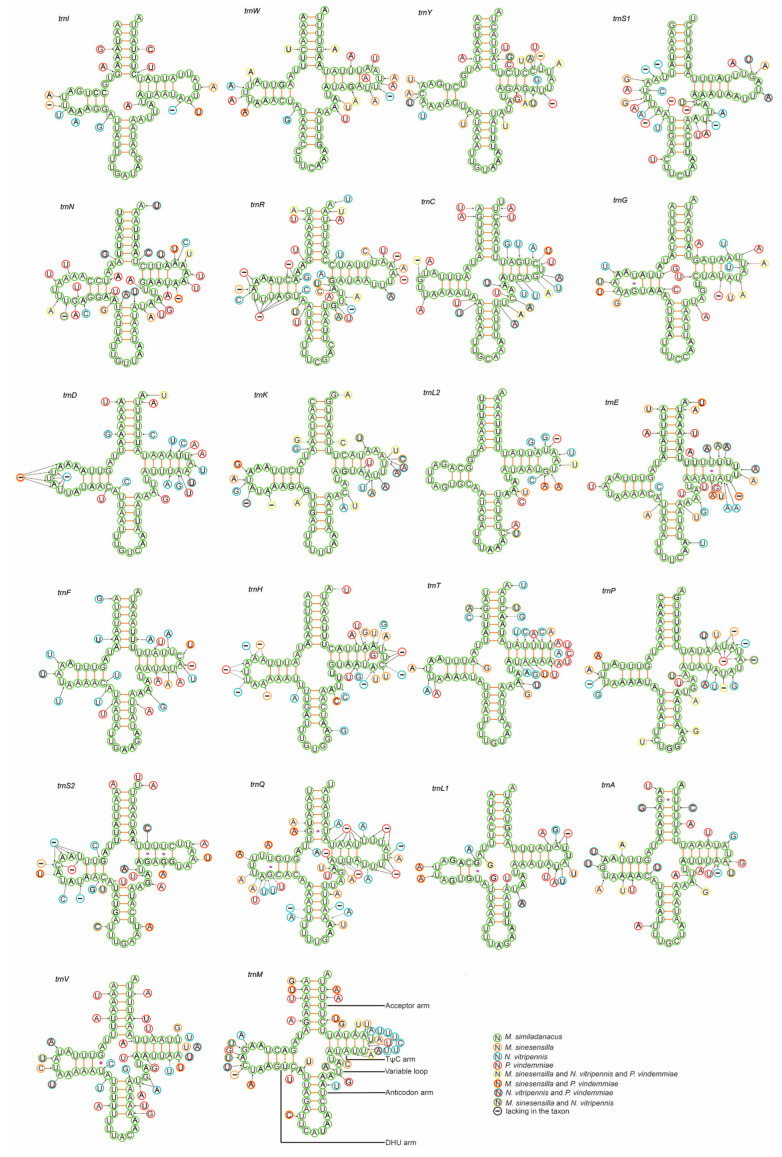
Predicted secondary cloverleaf structure for the tRNAs of *M. similadanacus*, *M. sinesensilla*, *N. vitripennis*, and *P. vindemmiae* of Pteromalidae.

In total, 22 tRNA genes were interspersed throughout the mitochondrial genomes of Pteromalidae. Within the tRNA secondary structures of pteromalid mitogenomes, the DHU arm of trnS1 was missing, while in *P. vindemmiae*, the DHU arm and TΨC arm were missing in *trnR* and *trnQ*, respectively. Many insects lack DHU arms in the *trnS1* secondary structures, while other secondary structures are typical clover structures. This folding probably happened early in the evolution of Metazoa [65]. The TΨC arm of some insects is also missing [66]. Among the 22 tRNAs, 16 tRNAs were in the J chain and the six others were in the N chain. Additionally, the size of tRNAs ranged from 55 to 74 bp (Figure 2). The average nucleotide composition of these tRNAs was A: 44.5%, T: 43.7%, C: 6.9%, and G: 4.9%, with a total average A + T content of 88.2%. Most AT skews were positive, except in *P. vindemmiae* (−0.006). In the same manner, all GC skews were negative, which indicates a slight bias towards the use of A and T in tRNAs.

The positions of large and small ribosomal RNA genes (*rrnL* and *rrnS*) were consistent with those observed in most other insects (between *trnL1-trnA* and *trnA-trnV*). The lengths of *rrnS* and *rrnL* for *N. vitripennis* were the longest among the four species, and the lengths of *rrnS* and of *rrnL* for *P. vindemmiae* were the shortest. Among these four species, the length of *rrnS* ranged from 763 to 787 bp, and the average AT content was 87.6% (*P. vindemmiae* had the highest AT content); the length of *rrnL* ranged from 1300 to 1325 bp, and the average AT content was 87.0%. In *P. vindemmiae*, the length of the *rrnS* gene was 763 bp with an AT content of 88.9%, while the *rrnL* gene was 1300 bp with an AT content of 87.5%.

### 3.5. Protein-Coding Genes

In our four new mitogenomes, 10 of 13 protein-coding genes were encoded by the majority strand, while 3 genes (*nad2*, *nad6* and *cytb*) were encoded by the minority strand; the length of the 13 protein-coding genes ranged from 161 bp to 1675 bp. Among them, the length of the 13 protein-coding genes of *P. vindemmiae* was 159-1672 bp. Among the 13 protein-coding genes, the shortest and longest encoding genes were *atp8* and *nad5*. In *M. similadanacus* and *M. sinesensilla*, the protein-encoding genes had the same length excepted for *nad1*, *nad5*, and *nad6*. The length of *cox3*, *atp6*, and *nad4l* were the same in the four newly sequenced species, being, respectively, 786 bp, 675 bp, and 288 bp.

The total length of the PCGs of the four new mitogenomes ranged from 11092 bp to 11117 bp. The shortest was 11092 bp in *P. vindemmiae* and accounted for 74.69% of the entire genome. The highest average AT content of the 13 protein-coding genes was 84.20% in *P. vindemmiae* and the lowest was 81.70% in *N. vitripennis*. In all new mitogenomes, *cox1* had the lowest AT content in the 13 PCGs, ranging from 76.9% (*P. vindemmiae*) to 74.5% (*N. vitripennis*), and *atp8* had the highest AT content in these 13 PCGs, ranging from 92.50% (*P. vindemmiae*) to 88.10% (*N. vitripennis*).

The preferred initiation codon of the pteromalid mitogenomes was ATN, as observed in most other insect mitogenomes [47], except for *nad1* of *N. vitripennis*, which starts with TTG. In these four pteromalid species, the start codon of six genes (*cox3*, *atp6*, *cox1*, *nad4*, *nad6*, and *cytb*) was ATG and it was ATT for the other three genes (*cox2*, *nad5*, *nad4l*). The stop codon of eight genes (*nad2*, *cox3*, *atp6*, *atp8*, *cox2*, *nad4l*, *nad6*, *cytb*) was TAA. TAG was the stop codon of *nad5* of *N. vitripennis*. Four genes of *P. vindemmiae* (*nad3*, *cox1*, *nad4*, and *nad5*), two genes of *N. vitripennis* (*cox1* and *nad4*), two genes of *M. sinesensilla* (*nad5* and *nad1*), and one gene of *M. similadanacus* (*nad5*) use the incomplete T- as stop codons (Table 2 and Table 3).

**Figure 3:**
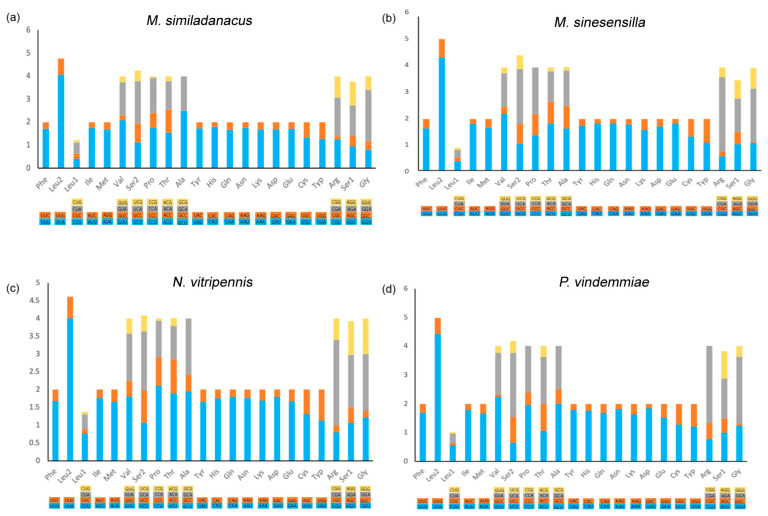
Relative synonymous codon usage (RSCU) of protein-coding genes of *M. similadanacus*, *M. sinesensilla*, *N. vitripennis*, and *P. vindemmiae*. (**a**) The RSCU of protein-coding genes of *M. similadanacus.* (**b**) The RSCU of protein-coding genes of *M. sinesensilla.* (**c**) The RSCU of protein-coding genes of *N. vitripennis.* (**d**) The RSCU of protein-coding genes of *P. vindemmiae*.

Codons with high AT content were preferred, which was consistent with most other insect mitogenomes [67]. The relative synonymous codon usages (RSCU) of these four species are shown in Figure 3. Taken together, the most frequently used codons were UUA (Leu2), CGA (Arg), GGA (Gly), UCA (Ser2), CCU (Pro), GUU (Val), and GCU (Ala), whereas codons ending with G or C, CUG, CUC, CAG, and GGC, were the less frequently used codons. The third codon position of A/T occurred more frequently than that of G/C, reflecting AT nucleotide bias in the mitochondrial PCGs among Pteromalidae.

### 3.6. Evolutionary Rate Analysis

The non-synonymous/synonymous substitution ratio (Ka/Ks) can be used to estimate whether a sequence is undergoing purifying, neutral, or positive selection. Among the four new mitogenomes, the Ka/Ks value of *cox1* was the lowest, at 0.12, 0.13, 0.14, and 0.14, respectively; among *M. sinesensilla* and *P. vindemmiae*, the Ka/Ks value of *atp8* was the highest at 0.97 and 1.59 respectively, but the highest Ka/Ks value of *M. similadanacus* was that of *nad2* (0.76), and the highest of *N. vitripennis* was that of *nad4l* (0.96).

**Figure 4:**
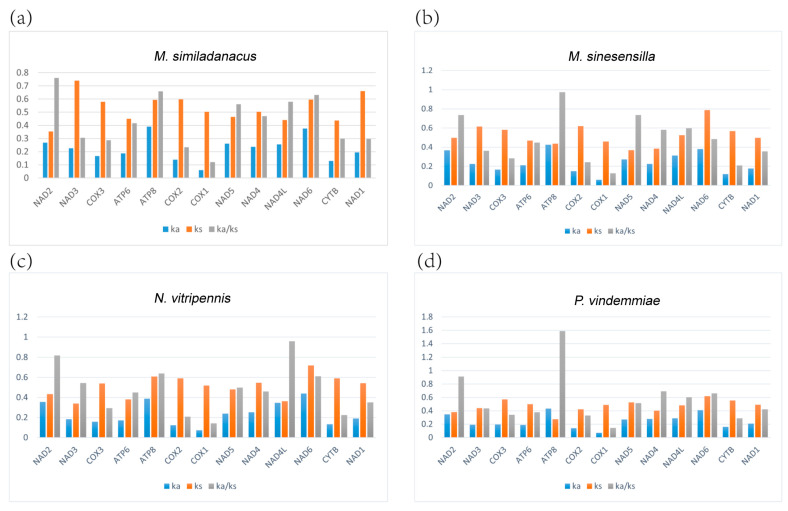
Evolutionary rates of mitochondrial genomes of Pteromalidae. The numbers of nonsynonymous substitutions per nonsynonymous site (Ka), the number of synonymous substitutions per synonymous site (Ks), and the ratio of Ka/Ks for every mitochondrial genome are given, using that of *Pachyneuron aphidis* as a reference sequence. (**a**) The Ka/Ks of *M. similadanacus.* (**b**) The Ka/Ks of *M. sinesensilla.* (**c**) The Ka/Ks of *N. vitripennis.* (**d**) The Ka/Ks of *P. vindemmiae*.

In total, only the Ka/Ks value of *atp8* of *P. vindemmiae* was greater than one. A Ka/Ks greater than one indicates that this protein-coding gene has been subjected to positive selection effects during evolution. This may suggest that non-synonymous mutations of *atp8* were selectively retained, which is rarely observed in insects. Following non-synonymous mutations, the mutated protein-coding gene was retained. Additionally, most of the 13 protein-coding genes were under purifying selection (Figure 4).

### 3.7. Phylogenetic Analysis

**Figure 5:**
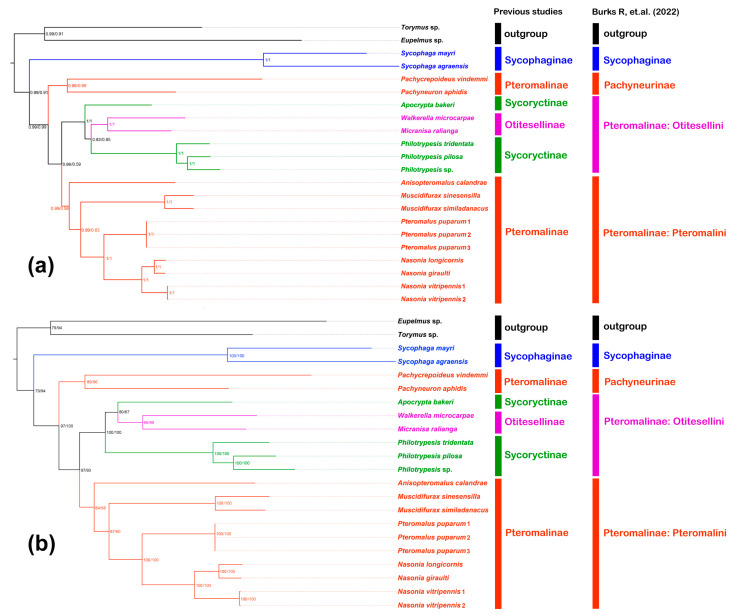
ML and BI phylogenetic trees based on the nucleotide sequence data of PCG12 and amino acid sequence data of AA: (**a**) represents the BI tree; Bayesian posterior probabilities (PP) are indicated on the branch, with values of AA at the left side and PCG12 at the right side; (**b**) represents the ML tree; ultrafast bootstrap (UFB) is indicated on the branch, with values of AA at the left side and PCG12 at the right side [68].

Two datasets (PCG12 and AA) and two inference methods, namely maximum-likelihood (ML) and Bayesian inference (BI), were used to reconstruct the phylogeny of pteromalid mitogenomes, and four topologies of ML-AA, ML-PCG12, BI-AA, and BI-PCG12 were reconstructed. All topologies were highly supported and both methods yield similar topologies. All pteromalid subfamilies were recovered as monophyletic. In all topologies, Pachyneurinae was recovered as sister to Pteromalinae, and this clade was then sister to Sycophaginae. Pteromalinae were subdivided into two monophyletic tribes, Otitesellini and Pteromalini (Figure 5).

### 3.8. Ancestral Character Reconstruction and Gene Rearrangement

**Figure 6:**
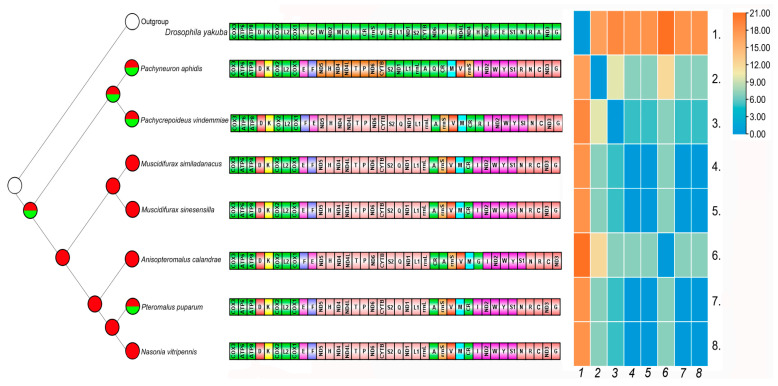
Left side is the ancestral state reconstruction based on the BI tree. Red pies represent primary parasitoids, half-green and half-red pies represent primary parasitoids and hyperparasitoids, and white pies represent undefined biology. Gene orders were arranged on the right side of pteromalids. Different gene rearrangements are highlighted in different colors. The heat map of the pairwise breakpoint distance of Pteromalidae is on the right side.

Based on the BI topology, the ancestral biology of Pteromalidae included in the analysis was inferred to be both primary parasitoid and hyperparasitoid (Figure 6).

Gene rearrangement patterns in Hymenoptera are usually more complex and variable than in other insect orders. Compared to the putative ancestral pattern [69,70] of the insect mitogenome, dramatic gene rearrangements were observed in Pteromalidae mitogenomes. These rearrangements occurred in tRNA genes but also in protein-coding genes. Five gene blocks were found in Pteromalidae (only considering complete mitogenomes). The first gene block was *cox3-atp6-atp8*, which existed in all sequenced species. With the exception of *Philotrypesis tridentata*, all other species shared the gene block *cox2*-*trnL2*-*cox1*. All species also shared the gene block of *nad5-trnH-nad4-nad4l-trnT-trnP-nad6-cytb*, but *P. tridentata* showed an inversion segment (*cytb-nad6-trnP-trnT-nad4l-nad4-trnH-nad5*). Finally, most species exhibited a gene block of *trnS2-trnQ-nad1-trnL1-rrnL-trnA*. In addition, gene block *trnI-nad2-trnW-trnY-trnS1* was shared by all sequenced genomes.

*Muscidifurax similadanacus*, *M. sinesensilla*, and *N. vitripennis* shared the same gene rearrangement pattern. Eighteen rearrangements were observed between these three pteromalid species and the putative ancestral mitogenome of *D. yakuba,* including one inversion from *trnV-rrnS* to *rrnS-trnV*, and three reversed genes. *Pachycrepoideus vindemmiae* exhibited a new rearrangement pattern in Pteromalidae and a total of 19 genes were rearranged (Figure 6). Compared with the gene order of *N. vitripennis*, three kinds of rearrangements were discovered in *P. vindemmiae* and one inversion from *trnE-trnF* to *trnF-trnE*, and trnR had a transposition; for details see Figure 6. In the mitogenome of *P. vindemmiae*, three gene blocks (*trnF-trnE*, *trnS1-trnN*, and *rrnS-trnV*) were shared with the putative ancestral *D. yakuba*. Comparison of *P. aphidis* and *P. vindemmiae* mitogenomes showed two (*CR-trnM-trnV-rrnS* and *trnE-trnF*) inversions and two (*trnQ and trnR*) transpositions. Comparison of *P. aphidis* and *A. calandrae* mitogenomes, highlighted three (*trnE-trnF*, *trnR-trnN*, and *trnV-rrnS*) inversions and two (*CR* and *trnG*) transpositions.

Comparison with other pteromalid mitogenomes revealed a novel inversion in the *trnE-trnF* gene cluster of Pachyneurinae and a novel transposition of *trnR* in *P. vindemmiae*, which was the first of this kind to be reported in Pteromalidae (Figure 6). The accelerated rate of gene rearrangement may be the result of the fast evolution of this group [71,72,73].

In Pteromalidae, most species have a high frequency of mitochondrial gene rearrangements, and the breakpoint distances range from 0 to 21 (Figure 6). According to our phylogeny, *M. similadanacus* and *M. sinesensilla* were the most closely related species, and the pairwise breakpoint distance between them was 0, which is in agreement with the assumption that a lower value of the pairwise breakpoint distance indicates close relationship in the topology (Figure 6). *P. vindemmiae* was sister to *P. aphidis* and the pairwise breakpoint distance between them was 10. *P. vindemmiae* and *M. similadanacus* were more distantly related in the topology; however, the pairwise breakpoint distance observed between them was only 5, which is a lower value than that observed between *P. vindemmiae* and *P. aphidis*. *N. vitripennis* and *A. calandrae* were closely related on the phylogenetic tree, and the pairwise breakpoint distance value was 7, whereas the value of *N. vitripennis* and *P. vindemmiae* was only 5. Globally, the pairwise breakpoint distance of mitogenome rearrangements appeared not to be consistent with the relationships and the proximity between Pteromalidae species observed in our topology.

## 4. Discussion

Gene rearrangement information is highly valuable for phylogenetic reconstruction of specific lineages [8,64], especially in the classification of low-level elements [27,74]. Gene rearrangement has been reported in most insect orders and an increased rate of gene rearrangements has been observed in Hemiptera [10,73], Protura [75], and Hymenoptera [4,14,62]. Since the pioneering work of Boore et al. [2], it has been acknowledged that the gene structure of mitochondrial genomes contains phylogenetic signal [3]. Detection of gene rearrangements in lower-level elements of insect mitogenomes is expected to shed light on the evolution of these taxa [62,74]. The mitochondria of Hymenoptera generally exhibit extremely high molecular evolution rates and extensive gene rearrangements due to their parasitic lifestyles. However, our understanding of the mitochondrial genome of pteromalids is still limited, and the mechanism of gene rearrangement is still unclear.

Our study confirms the gene block *cox3-atp6-atp8-trnD-trnK-cox2-trnL2-cox1* observed in Chalcidoidea and acknowledged by Wu et al. [16], though it was not observed in *P. tridentata*. Wu et al. [16] also observed a large conserved gene block (*nad5-trnH-nad4-nad4l-trnT-trnP-nad6-cytb-nad1-trnL1-rrnL*) in most mitogenomes of chalcidoid wasps. This gene block was also observed in most species of our study, except in *Philotrypesis*. Our study also corroborated the fact that most chalcidoid wasps have a gene cluster (*nad3-trnG*) next to *cox3*, except *Philotrypesis Pilosa,* as proposed by Wu et al. [16]. Two other gene blocks proposed by Wu et al. [16], *trnI-nad2-trnW* and *trnY-trnS1*, were found to be merged into a larger gene block (*trnI-nad2-trnW-trnY-trnS1*) in our new mitogenomes. Feng et al. [76] proposed that the gene block *CR-trnI-trnQ-trnM-nad2-trnW-trnC-trnY* was the main hot spot of gene rearrangement. Our study confirms that the gene rearrangement hot spot of Pteromalidae also occurs in this gene region (Figure 6).

The site-heterogeneous mixture model (CAT+GTR) implemented in PhyloBayes has been shown to correct the wrong grouping of unrelated taxa that share a similar base composition and an accelerated evolutionary rate [77]. ML and BI analyses yield similar topologies (Figure 5). Because Pteromalidae mitogenomes show a high evolutionary rate, we selected the BI topology to reconstruct the ancestral states. In previous classification, *Walkerella microcarpae* and *Micranisa ralianga* belonged to Otitesellinae and *Apocrypta bakeri*, *Philotrypesis tridentata*, *Philotrypesis pilosa*, and *Philotrypesis* sp. to Sycoryctinae. Based our results, Otitesellinae were recovered as nested within Sycoryctinae, which made Sycoryctinae paraphyletic, a result consistent with Zhao et al. [40] and Cruaud et al. [78]. The clade comprising Otitesellinae and Sycoryctinae appeared sister to other Pteromalinae included in our analysis, a result consistent with Rasplus et al. [23]. In addition, Sycoryctinae were not recovered as monophyletic, a result consistent with Munro et al. [18]. However, according to the classification system recently proposed by Burks et al. [68] and based on the analyses by Cruaud et al. [78], our results also corroborated that members of the previous Otitesellinae and Sycoryctinae belong in fact to Pteromalinae and represent at most a tribe of this subfamily. All other pteromalid subfamilies were recovered as monophyletic. Therefore, our study supports the classification proposed by Burks et al. [68] based on nuclear Ultra-Conserved Elements and exons [78]. Our result shows the power of mitogenomes to reconstruct family-level phylogenies in Chalcidoidea, only for three of the eight pteromalid subfamilies and 11 of the 500+ recognized genera. The monophyly of Pteromalidae was not directly tested in the present study and this will be an important issue for future studies.

The pairwise breakpoint distances of mitogenome rearrangements appeared not to be completely consistent with the relationships observed among our Pteromalidae species; see Figure 6. However, the number of mitogenomes used in this study is still limited and increasing sampling is necessary to confirm this result.

Our studies highlighted new Pteromalidae gene rearrangements, revealed the evolutionary relationship between the main groups of Pteromalidae, accumulated molecular data for the study of Pteromalidae phylogeny, and provided a genetic background for biological control in agriculture and forestry.

## Figures and Tables

**Table 1:** List of species investigated and their related information.

No.	Family	Subfamily	Tribe	Taxa	GenBank Accession No.	Location/Reference
1	Eupelmidae			*Eupelmus* sp.	MG923493	[27]
2	Torymidae			*Torymus* sp.	MG923516	[27]
3	Pteromalidae	Pachyneurinae		*Pachycrepoideus vindemmiae*	MT712142	This study, Anhui
4				*Pachyneuron aphidis*	MK577639	[16]
5		Pteromalinae	Pteromalini	*Anisopteromalus calandrae*	MW817149	Unpublished
6				*Muscidifurax sinesensilla*	MT712140	This study, Xinjiang
7				*Muscidifurax similadanacus*	MT712139	This study, Xinjiang
8				*Nasonia giraulti*	EU746611, EU746614	[26]
9				*Nasonia longicornis*	EU746612, EU746616	[26]
10				*Nasonia vitripennis*	EU746609, EU746613	[26]
11				*Nasonia vitripennis*	MT712141	This study, Shandong
12				*Pteromalus puparum*	MH051556	Unpublished
13				*Pteromalus puparum*	NC039656	Unpublished
14				*Pteromalus puparum*	MG923513	[27]
15			Otitesellini	*Apocrypta bakeri*	MT906648	[39]
16				*Micranisa ralianga*	MW167115	[40]
17				*Philotrypesis tridentata*	MT947602	[39]
18				*Philotrypesis pilosa*	JF808723	[14]
19				*Philotrypesis* sp.	JF808722	[14]
20				*Walkerella microcarpae*	MW167116	[40]
21		Sycophaginae		*Sycophaga agraensis*	MT947599	[39]
22				*Sycophaga mayri*	MW167114	[40]

**Table 2:** Mitogenomic organization of *M. similadanacus*, *M. sinesensilla*, and *N. vitripennis*.

	Position		Size (bp)	Intergenic Nucleotides	Codon		Strand
	From	To			Start	Stop	
*M. similadanacus/M. sinesensilla/N. vitripennis*							
trnI	1/1/1	69/68/70	69/68/70				+/+/+
nad2	89/87/92	1096/1094/1102	1008/1008/1011	19/18/21	ATT/ATT/ATA	TAA/TAA/TAA	−/−/−
trnW	1095/1101/1105	1161/1167/1171	67/67/67	−2/6/2			−/−/−
trnY	1160/1166/1170	1226/1230/1237	67/65/68	−2/−2/−2			+/+/+
trnS1	1233/1240/1240	1295/1302/1299	63/63/60	6/9/2			+/+/+
trnN	1299/1324/1302	1366/1391/1368	68/68/67	3/21/2			−/−/−
trnR	1362/1393/1375	1430/1463/1445	69/71/71	−5/1/6			+/+/+
trnC	1433/1475/1448	1496/1537/1514	64/63/67	2/11/2			+/+/+
nad3	1500/1537/1514	1850/1887/1864	351/351/351	3/−1/−1	ATT/ATT/ATA	TAA/TAA/TAA	+/+/+
trnG	1851/1888/1865	1916/1954/1932	66/67/68				+/+/+
cox3	1917/1960/1935	2702/2745/2720	786/786/786	−/5/2	ATG/ATG/ATG	TAA/TAA/TAA	+/+/+
atp6	2702/2745/2723	3376/3419/3397	675/675/675	−1/−1/2	ATG/ATG/ATG	TAA/TAA/TAA	+/+/+
atp8	3367/3410/3391	3528/3571/3549	162/162/159	−10/−10/−7	ATT/ATT/ATA	TAA/TAA/TAA	+/+/+
trnD	3529/3572/3551	3597/3636/3615	69/65/65	−/−/1			+/+/+
trnK	3601/3652/3619	3669/3722/3692	69/71/74	3/15/3			−/−/−
cox2	3670/3726/3696	4344/4400/4364	675/675/669	−/3/3	ATT/ATT/ATT	TAA/TAA/TAA	+/+/+
trnL2	4345/4401/4365	4410/4468/4432	66/68/68				+/+/+
cox1	4413/4471/4433	5942/6000/5963	1530/1530/1531	2/2/−	ATG/ATG/ATG	TAA/TAA/T	+/+/+
trnE	5945/6003/5964	6010/6067/6033	66/65/70	2/2/−			−/−/−
trnF	6009/6072/6045	6072/6136/6109	64/65/65	−2/4/11			+/+/+
nad5	6073/6137/6109	7741/7808/7794	1669/1672/1686	−/−/−1	ATT/ATT/ATT	T/T/TAG	+/+/+
trnH	7750/7812/7795	7818/7877/7859	69/66/65	8/3/−			+/+/+
nad4	7819/7879/7860	9159/9219/9198	1341/1341/1339	−/1/−	ATG/ATG/ATG	TAA/TAA/T	+/+/+
nad4l	9153/9213/9192	9440/9500/9479	288/288/288	−7/−7/−7	ATT/ATT/ATT	TAA/TAA/TAA	+/+/+
trnT	9441/9501/9479	9504/9564/9543	64/64/65	−/−/−1			−/−/−
trnP	9507/9565/9544	9575/9629/9608	69/65/65	2/−/−			+/+/+
nad6	9581/9633/9617	10126/10184/10165	546/552/549	5/3/8	ATG/ATG/ATG	TAA/TAA/TAA	−/−/−
cytb	10126/10185/10167	11271/11330/11306	1146/1146/1140	−1/−/1	ATG/ATG/ATG	TAA/TAA/TAA	−/−/−
trnS2	11271/11329/11305	11339/11396/11371	69/68/67	−1/−2/−2			−/−/−
trnQ	11341/11409/11398	11408/11480/11468	68/72/71	1/12/26			+/+/+
nad1	11409/11484/11469	12338/12405/12401	930/922/933	−/3/−	ATT/ATT/TTG	TAA/T/TAA	+/+/+
trnL1	12339/12406/12402	12404/12472/12468	66/67/67				+/+/+
rrnL	12405/12473/12469	13717/13780/13793	1313/1308/1325				+/+/+
trnA	13718/13781/13794	13782/13845/13857	65/65/64				+/+/+
rrnS	13783/13846/13858	14558/14625/14644	776/780/787				+/+/+
trnV	14559/14626/14645	14623/14691/14712	65/66/68				+/+/+
trnM	14626/14694/14716	14690/14758/14787	65/65/72	2/2/3			+/+/+
AT-rich	14691/14759/14788	15080/15020/14791	390/262/4				-/-/-

**Table 3:** Mitogenomic organization of *P. vindemmiae*.

	Position		Size (bp)	Intergenic Nucleotides	Codon		Strand
	From	To			Start	Stop	
*P. vindemmiae*							
trnR	1	59	59				−
trnI	63	131	69	3			+
nad2	167	1168	1002	35	ATT	TAA	−
trnW	1172	1241	70	3			−
trnY	1240	1305	66	−2			+
trnS1	1307	1366	60	1			+
trnN	1370	1438	69	3			+
trnC	1440	1504	65	1			+
nad3	1505	1853	349		ATT	T	+
trnG	1854	1918	65				+
cox3	1919	2704	786		ATG	TAA	+
atp6	2704	3378	675	−1	ATG	TAA	+
atp8	3372	3530	159	−7	ATT	TAA	+
trnD	3544	3608	65	13			+
trnK	3618	3689	72	9			−
cox2	3691	4359	669	1	ATT	TAA	+
trnL2	4360	4425	66				+
cox1	4426	5959	1534		ATG	T	+
trnF	5958	6020	63	−2			−
trnE	6021	6085	65				+
nad5	6086	7757	1672		ATT	T	+
trnH	7760	7823	64	2			+
nad4	7824	9156	1333		ATG	T	+
nad4l	9150	9437	288	−7	ATT	TAA	+
trnT	9437	9506	70	−1			−
trnP	9514	9577	64	7			+
nad6	9581	10,147	567	3	ATG	TAA	−
cytb	10,147	11,283	1137	−1	ATG	TAA	−
trnS2	11,282	11,348	67	−2			−
trnQ	11,364	11,418	55	15			+
nad1	11,420	12,340	921	1	ATT	TAA	+
trnL1	12,341	12,405	65				+
rrnL	12,406	13,705	1300				+
trnA	13,706	13,770	65				+
rrnS	13,771	14,533	763				+
trnV	14,534	14,600	67				+
trnM	14,601	14,667	67				+
AT-rich	14,668	14,850	183				-

**Table 4:** Nucleotide composition and skewness of mitogenomes.

Regions	Species	Size (bp)	T%	C%	A%	G%	AT (%)	GC (%)	AT Skew	GC Skew
Full genome	*M. similadanacus*	15080	41.0	9.0	42.5	7.5	83.5	16.5	0.018	−0.091
	*M. sinesensilla*	15020	40.7	8.8	43.4	7.1	84.1	15.9	0.032	−0.107
	*N. vitripennis*	14791	39.6	9.4	43.5	7.5	83.0	17.0	0.047	−0.112
	*P. vindemmiae*	14850	41.1	8.2	44.2	6.6	85.3	14.7	0.036	−0.108
PCGs	*M. similadanacus*	11107	39.7	9.3	42.5	8.4	82.3	17.7	0.034	−0.051
	*M. sinesensilla*	11108	39.7	9.1	43.2	8.1	82.8	17.2	0.042	−0.058
	*N. vitripennis*	11117	38.6	9.9	43.1	8.4	81.7	18.3	0.055	−0.082
	*P. vindemmiae*	11092	40.0	8.6	44.2	7.3	84.2	15.8	0.050	−0.082
tRNAs	*M. similadanacus*	1467	44.0	6.5	44.1	5.3	88.1	11.9	0.001	−0.102
	*M. sinesensilla*	1464	43.7	6.6	44.9	4.8	88.6	11.4	0.014	−0.158
	*N. vitripennis*	1484	42.2	7.7	44.9	5.2	87.1	12.9	0.031	−0.194
	*P. vindemmiae*	1438	44.7	6.7	44.2	4.4	88.9	11.1	−0.006	−0.207
rRNAs	*M. similadanacus*	2089	45.4	7.6	42.2	4.8	87.6	12.4	−0.037	−0.226
	*M. sinesensilla*	2088	44.3	8.1	43.4	4.2	87.7	12.3	−0.010	−0.317
	*N. vitripennis*	2112	43.1	8.0	43.8	5.0	86.9	13.1	0.008	−0.231
	*P. vindemmiae*	2063	44.8	7.5	43.2	4.5	88.0	12.0	0.018	−0.250
Control region	*M. similadanacus*	390	42.1	17.7	36.2	4.1	78.2	21.8	−0.075	−0.624
	*M. sinesensilla*	262	36.3	16.0	42.4	5.3	78.6	21.4	0.076	−0.502
	*N. vitripennis*	4	50.0	0.0	50.0	0.0	100.0	0.0	0.000	0.000
	*P. vindemmiae*	183	34.4	8.7	51.9	4.9	86.3	13.7	0.203	−0.279

## Data Availability

All sequences generated during this study have been deposited in the GenBank (https://www.ncbi.nlm.nih.gov/genbank/).
